# Uterine Polypus Treated by Injections of the Perchloride of Iron

**Published:** 1860-03

**Authors:** J. N. Graham

**Affiliations:** Chicago, Ill.


*Uterine Polypus treated by Injections of the Perchloride of Iron.* By J. N. GRAHAM, M.D., Chicago, Ill.

We notice in the November number of the AMERICAN MEDICAL MONTHLY an extract of a note from a previous number of the *Chicago Medical Journal*, entitled, "Nasal Polypus—New Treatment," and speaking of the effects of the injection of the muriated tincture of iron in cases of nasal polypi, and also of the possibility of its good results "in other localities less accessible to removal."

The note referred to put us in mind of a case of uterine polypus which came under our care in 1857, a brief account of which we take from our note-book, as follows:

June 20th, 1857, called to see Mrs. B.; found her very low and reduced by profuse hæmorrhage, occurring about the time, as she supposed, "of what ought to be the return of her regular monthly visitations." Previous to this, she had been very irregular in her menses, and "supposed it to be about the turn of life with her." She says the hæmorrhage has been very considerable; she is feeble, and depressed in spirits; pulse small, frequent, and tremulous; tongue covered with an ashy-white coat.

Put her upon an energetic course of treatment—tonics and astringents, with astringent injections, such as acetate of lead, tannin, sulph. zinc, &c. During the treatment we often introduced into the vagina portions of solid ice; also, bits of alum, enveloped in lint, were introduced into the os uteri. Notwithstanding this course of treatment, she continued, with but occasional and partial respite from the hæmorrhage, for between five and six days after we first saw her.

Becoming convinced that there must be some cause at the bottom of all this, other than the supposed "change of life," which, as yet, we had not ascertained, we made as thorough an uterine examination as the condition of the uterus would allow, and detected a soft, fleshy substance—pendulous, apparently, from the fundus uteri. As the neck of the uterus remained rigid, and further dilatation was not practicable, ergot was given in small, repeated doses, as well to arrest the bleeding, as to expel, if possible, what seemed to be the cause of the trouble. Having continued the ergot for some ten or fourteen hours, with ice and astringent injections, and the hæmorrhage continuing—sometimes, after a partial cessation, returning in alarming quantities—all our previous efforts to arrest it having failed, we injected the muriated tincture of iron, diluted in one-third mucilage gum-arabic, into the os—continuing the opium, tonic mixture, and ergot during the night.

June 25th, A. M.—Found the patient more comfortable—the os so dilated that we introduced the hand into the uterus sufficient to enable us to remove several small pieces of semi-consistent, hepatized floculi, with some well-defined fibrinous substance, resembling, in part of its formation, the sirloin portion of beef, and quite strongly attached to the fundus of the uterus—somewhat larger in its body than a hen's egg, with a vermiform portion extending downward.

The dilatable condition of the uterus at this time, and the ease with which we grasped the tumor, enabled us to remove it without the use of instruments.

During the removal of this body there was considerable hæmorrhage, which was soon arrested by cold injections of alum-water.

It may seem to some that a solution so strong of muriat. tinct. ferri was rather a harsh application, and uncalled for under the premises. The *urgency* of the case, and the failure of the previous treatment to arrest the discharge, prompted us to resort to this treatment. And then even with the tinct. ferri mur., in its full strength, we are of the opinion that the lubricating secretion, thrown out from the mouth of the uterus, would soon form a shield around even this escharotic and prevent injury to the parts.

We are not aware that the muriated tincture of iron has been used in like cases, nor are we conscious of having been prompted to its use here from any other source than a knowledge of the nature of the medicine, and a previous failure to arrest, by any other means, the hæmorrhage that had threatened the life of our patient. Judging from our experience in this case, we should have no hesitancy in using the tinct. ferri, in cases of severe uterine hæmorrhage, either injected through the os or applied by sponge or lint. If, as in the case referred to in the AMERICAN MEDICAL MONTHLY, the iron was borne with impunity, injected against the delicate and sensitive nasal membrane, surely it would be tolerated by the much less sensitive uterus.

It will be remembered that, in the above report, though the ergot had been used for ten or more hours, in connection with the previous applications, the bleeding was not stopped, nor could the polypus be reached or removed.

We think the iron acted a favorable part in astringing or cauterizing the relaxed mouths of the bleeding vessels, and separating the attachments of the fungus. The patient rapidly recovered after this.

We report the above, hoping that, if based upon correct principles, the use of this agent, in like critical cases, adapted to the circumstances of the case, may prove useful in the hands of others.

While we would discard the reckless or indiscriminate use of caustics or escharotics, we are of the opinion that their application to the uterus is not fraught with that danger that by many has been supposed, nature threwing out a secretion (lubricating) that liquefies or envelops even the solid nitrate of silver, thus preventing it from further invasion.

